# The Foraging Ecology of the Mountain Long-Eared Bat *Plecotus macrobullaris* Revealed with DNA Mini-Barcodes

**DOI:** 10.1371/journal.pone.0035692

**Published:** 2012-04-24

**Authors:** Antton Alberdi, Inazio Garin, Ostaizka Aizpurua, Joxerra Aihartza

**Affiliations:** Department of Zoology and Animal Cell Biology, Faculty of Science and Technology, University of the Basque Country UPV/EHU, Sarriena z.g., Leioa, The Basque Country; University of Western Ontario, Canada

## Abstract

Molecular analysis of diet overcomes the considerable limitations of traditional techniques for identifying prey remains in bat faeces. We collected faeces from individual Mountain Long-eared Bats *Plecotus macrobullaris* trapped using mist nets during the summers of 2009 and 2010 in the Pyrenees. We analysed their diet using DNA mini-barcodes to identify prey species. In addition, we inferred some basic features of the bat's foraging ecology that had not yet been addressed. *P. macrobullaris* fed almost exclusively on moths (97.8%). As prey we detected one dipteran genus (Tipulidae) and 29 moth taxa: 28 were identified at species level (23 Noctuidae, 1 Crambidae, 1 Geometridae, 1 Pyralidae, 1 Sphingidae, 1 Tortricidae), and one at genus level (*Rhyacia* sp., Noctuidae). Known ecological information about the prey species allowed us to determine that bats had foraged at elevations between 1,500 and 2,500 m amsl (above mean sea level), mostly in subalpine meadows, followed by other open habitats such as orophilous grasslands and alpine meadows. No forest prey species were identified in the diet. As 96.4% of identified prey species were tympanate moths and no evidence of gleaning behaviour was revealed, we suggest *P. macrobullaris* probably forages by aerial hawking using faint echolocation pulses to avoid detection by hearing moths. As we could identify 87.8% of the analysed sequences (64.1% of the MOTUs, Molecular Operational Taxonomic Units) at species level, we conclude that DNA mini-barcodes are a very useful tool to analyse the diet of moth-specialist bats.

## Introduction

The study of the trophic resources used by a species and the habitats where they are consumed are key aspects of addressing foraging ecology, which will provide a basic understanding of the relationships among consumers, resources, and environment [1]. Elucidating diet and habitat preferences is, therefore, paramount for any management or conservation purposes. Nevertheless, such studies are often beset by problems such as difficulty in obtaining information on elusive animals, capture/handling restrictions imposed by conservation status, or other methodological constraints. Thorough investigations of diet may provide sufficient information on most animals' foraging requirements, but such detailed estimates have hitherto been hard to achieve due to limitations in methods used.

Bat diet studies began with analysis of prey remains collected under feeding perches (e.g. [2], [3]) and inspection of stomach contents from sacrificed bats [4], [5]. The former technique digs out mostly the biggest prey [6], i.e. those dismembered prior to ingestion, and is limited to bat species that use perches and to individuals with known perching sites. The latter method entails sacrifice of animals that usually belong to protected species, and it is no longer used.

In recent decades, most dietary studies on bats have been carried out through morphological identification of prey fragments enduring in faeces. This approach depicts the diet realistically [7], [8], and it has allowed researchers to analyse allocation of food resources among separate bat species [9]–[11] or to study prey selection through comparison of diet and prey abundance in foraging areas (e.g. [12], [13]). In fact, a comprehensive feeding ecology of nearly all bat species has been described using this technique (e.g. [14]–[16]). Underrepresentation of soft-bodied prey is usually assumed, however, because their identifiable parts are less likely to persist [17]. Moreover, the key morphological features used to identify lower taxa are fatally damaged through digestion. Consequently, prey remains are seldom identified below the ordinal or family level [7], [18]. To increase resolving power, some authors have combined morphological identification with stable isotopic analysis of faeces [19], though they were only able to suggest the families of ingested items.

The development of molecular techniques has now taken the analysis of diet a qualitative step forward [20]. Along with the standardisation of a single molecular marker and the development of a large reference database (BOLD Systems, www.boldsystems.org; [21]), diet studies increasingly employ DNA barcodes [22]–[24]. The animal DNA barcode is a small fragment of the mitochondrial genome (COI gene) that is being sequenced in many species as an identity label [25], [26], which allows species-level description of an animal's diet.

Beyond a mere listing of prey, species-level identification affords the opportunity to test hunting-strategy hypotheses that could not previously be addressed with analysis techniques lacking the required resolution. For instance, the predator-prey relationship between bats and eared moths is one of the best exponents of coevolutionary arms race [27]. Although sensorial adaptations and related evasive and attacking behaviours have been extensively studied [28]–[35], strikingly, the actual contribution of eared moths to any bat species' diet has seldom been revealed to the family level [36]–[38]. Traditional techniques are generally unable to identify the species of consumed moths, so insight on precise predator-prey relationship could only be occasionally ascertained, mostly when culled parts were available [39]–[41]. Conversely, molecular identification of a tympanate moth as one of the main prey of *Barbastella barbastellus* [24] enabled an investigation showing that some aerial hawking bats hunt tympanate moths by casting faint echolocation calls that overcome the moths' hearing ability [42]. As many biological and environmental factors contribute to the diet of bats, molecular analysis of prey can also be a handy tool to spot a wide spectrum of habitat parameters. For example, Clare et al. [43] inferred ecosystem-level features by assessing the quality of water environments where bats fed through a quality ranking of the source environments of prey identified by molecular means.

The Mountain Long-eared Bat *Plecotus macrobullaris*, Kuzjakin 1965, was accepted as a species in 2002 [44]–[46] (Fig. S1). Most of the published research on the species has referred to its morphology or phylogenetics [47]–[51]. Its ecology is still poorly described, and its foraging preferences are controversial. Breeding colonies have been found in man-made buildings at 400–1,300 m in the Alps [51], [52] and as low as at sea level in Croatia [48]. *P. macrobullaris* has been captured by mist net in pastures above 1,800 m and as high as 2,800 m in the Pyrenees [53], [54] and in montane open areas with sparse shrubbery in the Middle East [50]. Accordingly, a recent study [51] documented the resemblance of the species' trophic niche to that of the open-land forager *P. austriacus* [55]. On the other hand, the echolocation and morphological characteristics of *P. macrobullaris* have prompted some authors to believe it is suited to forage in narrow rather than open spaces [51], [56]. This latter hypothesis seems to be supported by mist-net captures in Croatian forests and roost placements in Switzerland [48], [57]. Nevertheless, in a recently published radio-tracking study carried out in a nursery colony in Northern Italy bats avoided woods when foraging, selecting open rural areas and ecotones [58].

Recent studies on the diet of *P. macrobullaris* have revealed the importance of moths [16], [59], though the exact species composing its diet remain unidentified. Fortunately, Lepidoptera is one of the insect taxa with an extensive database of DNA barcodes available online (more than 70,000 species). Therefore, in this study we aimed to unveil the foraging behaviour of the Mountain Long-eared Bat *P. macrobullaris*. Particularly, we broadly tested the bat's alpine nature by investigating the elevational distribution and habitat of prey. Meanwhile, through detailed inspection of the prey species, we tested whether the Mountain Long-eared Bat feeds on hearing and non-flying prey. We used molecular tools to identify consumed prey at species level, and relied on available information about the habitats of moths and associated host plants. We have also assessed the usefulness and reliability of molecular tools in diet analysis.

## Materials and Methods

### Ethics Statement

Capture and handling protocols met the guidelines for treatment of animals in research and teaching [60], were approved by the Regional Council, and met Spanish legal requirements. The Government of Aragón (Spain) gave the necessary permits to carry out the fieldwork (LC/mp 24/2009/2958 and LC/ehv 25/2010/3234). Bats were released after being measured and faeces collected. To minimise stress, retention time never exceeded 30 minutes.

### Sample collection

Sampling was carried out during the summers of 2009 and 2010 in 25 different locations of the Pyrenees mountain range. We captured bats by mist nets placed at commuting paths, water points, and putative foraging areas 1,000–2,100 m amsl. Habitat types sampled were coniferous and holm oak forests (1,000–1,400 m, n = 6 sampling sites), subalpine meadows (1,500–1,900 m, n = 7), alpine meadows (1,700–2,100 m, n = 7), and rocky areas with poor vegetation cover (1,900–2,600 m, n = 5). In the Pyrenees, as in the Alps and many other European mountain ranges, the tree line has been lowered for pastoral activities by clearing subalpine shrubs and forests [61]–[64]. Consequently, the lower boundary of some alpine meadows in the Pyrenees has been lowered as much as 500 m.

Each trapped bat was held individually in a clean cloth bag for 10–25 min to collect faeces. Droppings were stored dry and frozen within 6 h of collection.

### DNA isolation and amplification

Each individual bat was considered as a sampling unit [65]. For each bat specimen, 20–40 mg of faeces (2–6 pieces of guano) were used for DNA extraction with the DNA Stool Mini Kit (Qiagen), following the manufacturer's instructions with some modifications [24].

Although a 650-bp sequence has been standardised as the molecular barcode [66], DNA degradation in droppings commonly forces the use of shorter sequences, even less than 200 bp [67]. Those short sequences perform at 90% species resolution in taxa with high species-level specificity [68], [69]. Moreover, their usefulness for prey identification in droppings has successfully been tested [24]. Consequently, a 157-bp fragment of COI gene was amplified using ZBJ-ArtF1c and ZBJ-ArtR2c primers developed by Zeale et al. [24]. The PCRs were performed using the Biotaq PCR kit (Bioline, www.bioline.com) in 10-µl-volume reactions. Each reaction contained 1.05 µl deionised water, 1 µl NH4×10 buffer, 0.4 µl 50 mM MgCl_2_, 1 µl 2 mM dNTPs, 0.75 µl 10 µM forward primer, 0.75 µl 10 µM reverse primer, 0.05 µl BIOTAQ DNA polymerase, and 5 µl of DNA sample extracted from the faeces. We used the PCR protocol for amplification developed by Zeale et al. [24]. PCR products were visualised on a 1.5% agarose gel to determine the amplicon∶primer-dimers ratio and choose the correct reaction products for cloning. PCR reactions were optimised to avoid the purification step of PCR-products, preventing DNA loss, and raising the ligation efficiency.

### DNA cloning and sequencing

The pGEM-T Easy Vector System and high-efficiency competent cells (≥1×10^8^ cfu/µg DNA) (Promega www.promega.com) were used for cloning the PCR products. A 3∶1 insert-to-vector molar ratio was used for ligation and reactions were carried out with overnight incubation at 4°C. Transformation was performed following the manufacturer's instructions. Twenty colony-forming units (cfu) were selected from each plate, and cloned inserts were liberated from cells and amplified using the universal M13 primers. Each PCR contained 20.8 µl deionised water, 2.5 µl NH4×10 buffer, 0.75 µl 50 mM MgCl_2_, 0.5 µl 10 mM dNTPs, 0.1 µl 0.2 mM M13 forward primer (5′-CCCAGTCACGACGTTGTAAAACG-3′), 0.1 µl 0.2 mM M13 reverse primer (5′-AGCGGATAACAATTTCACACAGG-3′), and 0.25 µl Taq DNA polymerase to complete a reaction volume of 25 µl. PCR thermal cycling conditions were as follows: 5 min at 94°C (longer than usual, to lyse bacteria), followed by 35 cycles of 45 s at 94°C, 45 s at 50°C, and 45 s at 72°C. The cycles ended with one final extension of 8 min at 72°C. Resulting amplicons were unidirectionally sequenced using M13 forward primer.

### Data analysis

The obtained sequences were aligned and edited with BioEdit [70], and deposited in the Dryad database (http://dx.doi.org/10.5061/dryad.611310kt). We used the software jMOTU [71] to collapse sequences into Molecular Operational Taxonomic Units (MOTUs) setting the threshold-value to 1.3% (2 nucleotides). This is less conservative than the threshold-values used by Bohmann et al. [23], Clare et al. [43] and Razgour et al. [72] –2.5%, 2.0% and 2.0% respectively–, but given the small sampling range we anticipated less diversity among available insects. The most common sequences from each MOTU were used for species identification. BLAST was used for sequence similarity analysis, comparing query sequences to reference sequences stored in GenBank derived from BOLD [21]. Taxonomic level thresholds were chosen based on our own data and bibliography [22], [43]. Species-level identification was assigned when similarity between query and reference sequences was greater than 98.7%, i.e. equal or less than 2 mutations. Similarities from 97.4% to 98.7% were identified at genus level. Similarities under 97.4% were not used to assign any sequence to a higher taxonomic level because they might be error prone [43]. As some MOTUs matched more than one species above 98.7% level, we gave different confidence levels to the identifications, based on the following criteria:

1. **Single match**: the sequence matches a single species with the selected similarity threshold (2 mutations),
2. **Several global/single local matches**: the sequence matches more than one species with the selected similarity threshold, but just one of them is present in Western Europe.
3. **Several global/several local matches**: the sequence matches more than one species present in Western Europe with the selected similarity threshold, but just one of them matches at 100%.

Rarefaction curves with 1,000 iterations were carried out for each sample with EstimateS 8.2.0 [73]. Dietary diversity was determined at the MOTU level using the Shannon's diversity index, where *p_i_* was defined as the number of samples in which MOTU *i* was found divided by sum of occurrences of all MOTUs [72].

### Prey item analysis

We used the relationship between body mass and wing-length in noctuid moths published by Wood et al. [74], to assess average body mass of consumed prey. Moths consumed by *P. macrobullaris* were grouped according to their elevational preference as follows: (1) widespread species occurring in a wide elevational range from sea level up to alpine meadows (0–2,800 m), (2) orophilous-alpine species appearing in both montane and alpine areas (600–2,500 m), or (3) strictly alpine species (>1,500 m).

Identified moth species were also classified according to their known foraging habitat preferences ([75]–[81] and www.nmnhs.com/butterfly_areas_bg/, accessed in April 2011). We distinguished 6 habitat types: (1) alpine meadows, grasslands extending up from the tree line, i.e. the imaginary line delimiting the highest elevations where trees at least 3 m tall are found in distinct patches [82]; (2) subalpine meadows, grasslands extending from the timberline to the tree line [82]; (3) orophilous grasslands, meadows below the timberline; (4) rockies, barren lands with vegetation cover less than 15% [83]; (5) shrublands, areas with shrub canopy (heath, juniper, etc.) greater than 20% of total vegetation; and (6) forests, either coniferous or deciduous woods.

## Results

**Figure 1:**
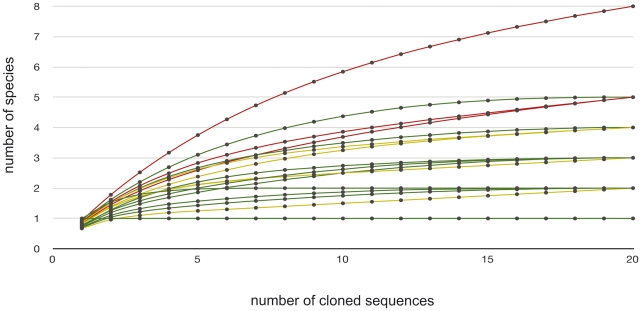
Diversity of prey sequences detected in samples in relation to number of clones sequenced. The rarefaction curves for individuals' samples showed that 22 out of 29 clearly reached the asymptote (green), four almost did (yellow), and three clearly did not (red), which means that the amount of clones sequenced per sample was high enough to detect the great majority of prey sequences in it.

In 12 out of the 25 sampled locations we captured 40 Mountain Long-eared Bats. DNA with sufficient quality for analysis was extracted from 31 of 40 individual faecal samples (77%), and at least 20 colonies were grown in 29 of those 31 (93%); thus, 102 faecal pellets produced by 29 individuals from 12 different locations were analysed. Rarefaction curves for individuals' samples showed that 22 out of 29 clearly reached the asymptote, 4 almost did, and 3 clearly did not (Fig. 1).

We analysed 20 colonies/sequences from each individual faecal sample, examining a total of 580 DNA sequences, among which we found 90 different sequences that were collapsed into 54 MOTUs. We identified 34 of the MOTUs to the likely species and 11 to genus level. All except one came from insects (Table 1): 44 in the Order Lepidoptera and one in the Order Diptera (Genus Tipula). Eight MOTUs could not be identified to species or genus level and the remaining one may correspond to bacteria, fungi, or another microbial element.

**Table 1:** Prey taxa detected in the diet of *Plecotus macrobullaris*.

Class	Order	Family	Genus/Species	Level	% Occur.	N sites
Insecta	Diptera	Tipulidae	*Tipula sp.*	1	3.4	1
	Lepidoptera	Crambidae	*Nomophila noctuella*	1	6.9	1
		Geometridae	*Nebula nebulata*	1	10.3	2
		Noctuidae	*Agrotis sp.*	-	10.3	2
			*Agrotis clavis*	1	31.0	5
			*Agrotis fatidica*	2	3.4	1
			*Agrotis simplonia*	1	6.9	2
			*Anarta odontites*	1	3.4	1
			*Apamea sp.*	-	10.3	2
			*Apamea furva*	1	3.4	1
			*Apamea lateritia*	1	13.8	4
			*Apamea maillardi*	2	17.2	2
			*Apamea monoglypha*	1	13.8	3
			*Apamea platinea*	1	3.4	1
			*Apamea sublustris*	1	6.9	2
			*Apamea zeta*	1	13.8	3
			*Autographa sp.*	-	3.4	1
			*Autographa gamma*	1	24.1	5
			*Chelis maculosa*	1	6.9	2
			*Dichagyris renigera*	2	3.4	1
			*Epipsilia grisescens*	2	6.9	2
			*Euxoa decora*	2	3.4	1
			*Hada plebeja*	2	3.4	1
			*Hadena compta*	3	10.3	2
			*Leucania comma*	2	3.4	1
			*Lycophotia porphyrea*	1	3.4	1
			*Mythimna conigera*	1	10.3	2
			*Noctua pronuba*	2	3.4	1
			*Rhyacia sp.*	-	3.4	1
			*Sideridis reticulata*	1	3.4	1
		Pyralidae	*Pempelia palumbella*	1	3.4	1
		Sphingidae	*Deilephila porcellus*	1	3.4	1
		Tortricidae	*Eana sp.*	-	3.4	1
			*Eana argentana*	1	3.4	1
		Unknown	8 unidentified MOTUs	.	3.4 each	1 each

**Level** refers to the confidence level of identification, namely: 1) Sequences matching a single species according to 98.7% of similarity-threshold, 2) Sequences matching more than one species (>98.7%), but only one of them being present in the Pyrenees, 3) Sequences matching more than one species in the Pyrenees (>98.7%), but only the shown species matches at 100%. **% Ocurr** gives the percentage of occurrence, i.e. the frequency of individual faecal samples in which the taxon has been identified. **N sites** indicates the number of sampling sites where the taxon was identified.

We successfully identified 28 moth species (539 sequences, 31 MOTUs) and one moth genus (*Rhyacia*) (Table 1). In some cases more than one different MOTU was assigned to the same species: three MOTUs were identified as *Agrotis simplonia*, and two as *Apamea zeta*; similarly, 11 MOTUs were linked to five genera, and some of them were subsequently identified at species level. Among the moths identified at species level, 27 species (23 Noctuidae, 1 Geometridae, 1 Crambidae, 1 Sphingidae, 1 Pyralidae) and one genus (Noctuidae) were tympanate lepidopterans, while one species (Tortricidae) was not; 98.1% of species-level-identified MOTUs belonged to eared moths, while 1.9% belonged to non-tympanate lepidopterans.

The average wingspan of prey moth species in the diet of the Mountain Long-eared Bat was 37.60 mm (SD ± 7.87, range 21–55). We calculated that *P. macrobullaris* feeds on moth species with an average body mass of 177 mg (SD ± 82, range 35–281).

The average number of prey species per bat was 2.75 (SD ± 1.6, range 1–8), more than 50% of the individuals had only one or two species in their faeces, and 28 of the 29 individuals showed five species or fewer, while the faeces of one exceptional bat contained 8 species. The average number of prey species per sampling site was 5.58 (SD ± 3.5, range 1–13). Shannon's index of dietary diversity was 3.76.

**Figure 2:**
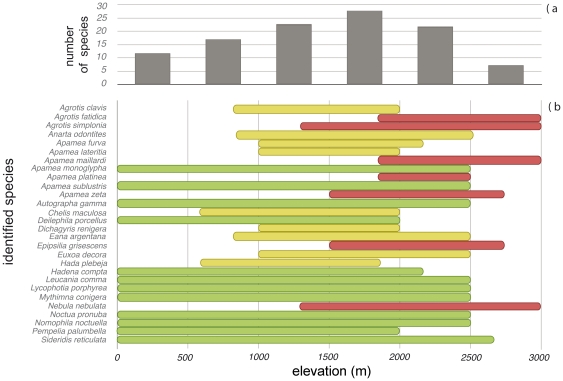
Elevational distribution of the moth prey species identified in faeces. a) Number of identified prey species present in each elevational range; b) elevational ranges of each prey identified at species level in South Europe. Species with wide elevational ranges are drawn in light green, orophilous-alpine species in yellow, and strictly alpine species in magenta. Bibliographic sources are given in the main text.

**Figure 3:**
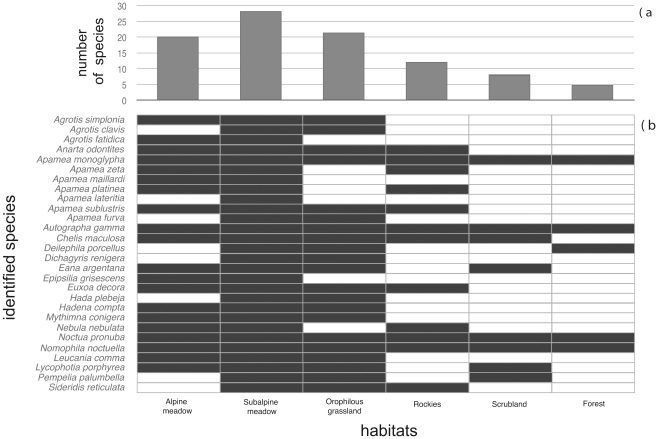
Foraging habitats of the prey species identified in faeces. a) Number of identified prey species foraging in each habitat type; b) habitat types used by each identified prey species in South Europe. Used habitats are drawn in black. Bibliographic sources are given in the main text.

The identified prey species occupy very diverse elevational ranges (Fig. 2). Strictly alpine species were found in 15 samples and orophilous-alpine species in 13 samples. Species with wide elevational ranges appeared together with strictly alpine species in five individuals, with orophilous-alpine species in four individuals, and alone in six. Most of the sequences belonged to species with broad elevation range (42.8%), followed by orophilous-alpine species (32.3%) and strictly alpine species (25.0%). The leading habitat types used by moths consumed by *P. macrobullaris* are subalpine meadows, followed by orophilous meadows and alpine meadows (Fig. 3). Thus, almost all the habitat types used by consumed moth species are open habitats. In addition, 76% of the detected moths use host plants from grasslands.

## Discussion

### Diet composition

As all but one of the MOTUs identified at species or genus level from the faeces of the Mountain Long-eared Bat belonged to the Order Lepidoptera, *P. macrobullaris* can be referred to as a moth specialist, in accordance with previous data reported across distant areas such as Turkey and the Alps [16], [59]. In this work, however, we were able to identify the species of moths composing its diet, following the path of recent studies that have also described the prey of bats by molecular techniques, achieving parallel taxonomic resolution [22], [24], [43], [72]. The most frequent prey species in our sample was *Agrotis clavis*, appearing in grasslands and open habitats of mountain areas [78], [81], which widely overlap the distribution range of *P. macrobullaris*. It was followed by *Apamea maillardi*, defined as arctic-alpine [84], and the ubiquitous species *Autographa gamma* and *A. monoglypha*. Other minor prey previously reported for *P. macrobullaris* –e.g. coleopterans and dipterans [59]–, were either absent or infrequent in the obtained sequences.

Although *Plecotus* species base their diet mainly on lepidopterans, and share common and ubiquitous prey species such us *Autographa gamma* or *Noctua pronuba* [72], there are several differences among them. The number of MOTUs (54), orders detected (2), the number of prey taxa per dropping (2.75) and the dietary diversity (3.76) detected in *P. macrobullaris* is smaller than in the case of *P. auritus* and *P. austriacus* [72]. This fact may be the result of two factors. On the one hand, high-mountain environments are generally less diverse than lowlands [85], so the results may reflect the available food diversity. On the other hand, the method (high throughput pyrosequencing) used by Razgour *et al.* [72] detects almost all the sequences present in the sample, while the cloning approach used by us may fail to detect the less abundant ones as it is constrained by the number of clones used for analysis of each sample.

### Alpine nature

*Plecotus macrobullaris* fed readily on prey found in subalpine and alpine elevational belts, which suggests that in summer they indeed forage between 1,500 and 2,500 m in the Pyrenees. In fact, all the prey species identified in this study have been reported to fly up to at least 1,800 m. Strictly alpine or subalpine prey species were detected in half of the samples. Moreover, the concurrent appearance in some faeces of moths with wide elevational ranges and those strictly alpine suggests that the former prey were also captured at high elevation. In fact, despite being more common in the lowland, some altitudinally ubiquitous moth species can often appear at higher elevations [86], [87]. Among the prey of *P. macrobullaris*, the moth *Autographa gamma* shows high migratory activity in Europe [73], [88], *Apamea monoglypha* has the migratory potential to cover large distances at high elevation [73], and the abundance of so-called “lowland moths” such as *Noctua pronuba* can be very high in alpine habitats as well [81], [86], [87]. Similarly, the altitudinally widely distributed *Hadena compta* is the main pollinator of the rock pink (*Dianthus sylvestris*), commonly found on rocky slopes at 1,000–2,800 m in the Alps [89], [90]. Additionally, most of the moth species identified as prey only appear in open areas, and more than 75% of their host plants are linked to meadows. We did not find any moth with strict forest habits, and only a handful of the generalist moths that appeared in faeces (*Apamea monoglypha, Noctua pronuba, Nomophila noctuella*) can be found in forests as well as in open areas. These findings are in accordance with the results obtained by Preatoni *et al.* [58], who observed that female Mountain Long-eared Bats avoid woods and make use of open areas and ecotones, showing a habitat use pattern more similar to *P. austriacus* than to *P. auritus*.

Although no bat species has been so far defined as purely alpine, a number of bats exploit high mountain environments [64], [91]–[93]. Alpine habitats are rich environments [94], [95] holding a great diversity of vertebrates and insects [87], [98]. Thus, moths linked to high-elevation meadows constitute a suitable resource for *P. macrobullaris*, which is likely most profitable when the highest meadows bloom as summer progresses. At the same time, exploiting this resource may help *P. macrobullaris* avoid and/or reduce interspecific competition with other moth specialists such as *P. austriacus* [59], [72] in lower open habitats.

However, the alpine habitat is known to exhibit extreme abiotic conditions regarding temperature, wind, solar radiation, and hypoxia [91], [96]–[98]. With low temperatures and a short vegetative growth period, some putative foraging areas of *P. macrobullaris* are covered with snow until June [82]. Hence, sprouting or blossoming and the matching phenology of insects are also delayed at high elevation, which, altogether, shortens the lifespan of adult phases [99]. In fact, most of the moths identified in this study only fly after June. Consequently, alpine habitats are suitable for bats for a short period that includes summer months only, as no moth species are found above 1,600 m until June at higher elevations [85]. Therefore, we should expect some differences in diet composition in spring, likely reflecting the bat's foraging at lower elevations, as do many other alpine species such as the Chamois *Rupycapra sp.* or the Alpine Accentor *Prunella collaris* [100], [101].

### Hunting

All the prey species identified in this study were nocturnal flying insects, and thus gleaning behaviour cannot be inferred for *P. macrobullaris*, in accordance with results recently published by Ashrafi et al. [59]. Further, almost all were tympanate moths, mostly of the family Noctuidae (23 out of 28). Rather than indicating any selective behaviour, these results likely reflect the actual prey availability at high elevation, because noctuids and geometrids are almost the only lepidopteran taxa present in alpine environments, the former eight times more abundant than the latter [85]. Moths of the families Noctuidae, Geometridae, Crambidae and Pyralidae, as well as Notodontidae, are able to hear ultrasounds [28], [102], [103]. That sensitivity allows the moths to detect and escape bats, and therefore reduces the risk of predation [37], [104]. To overcome this ability, the echolocation frequency of some moth-eating bats is displayed outside the sensitive frequency range of prey [31], [105]; but unlike them, long-eared bats of the genus *Plecotus* studied so far use low-intensity echolocation calls to avoid detection when approaching moths [106], [107]. Plausibly, *P. macrobullaris* may also emit faint echolocation signals when hunting by aerial hawking.

### Methodology

Mini-barcodes are a useful tool for studies with degraded DNA, such as diet analyses of bats. We could identify many prey sequences at species level, so the achieved taxonomic resolution considerably exceeds that attained by morphological analysis of faecal content, both in *P. macrobullaris* [16], [59] and in other moth specialists (e.g. [13], [108]).

Lepidopterans—all the sequences but one identified from faeces of *P. macrobullaris*—as well as coleopterans and orthopterans have yielded good identification success using DNA barcodes [109], [110]; in contrast, other taxa such as dipterans or hemipterans do not yield such effective results [111], [112]. Moths, beetles, grasshoppers, and crickets represent the bulk of food resources for many bat species, and hence, DNA barcodes will surely provide a great amount of information on these bats' ecology.

Nevertheless, there are some methodological limits worth mentioning. Although we lowered the threshold value for defining MOTUs comparing to other similar studies [23], [43], [72], anticipating less diversity among insects in high elevations, in 70% of the cases the achieved relationship among MOTUs and species was not 1∶1. This strongly contrasts the success or MOTU:species agreement observed by Razgour et al. [72]. In fact, several MOTUs (9.4%) were identified as belonging to the same species suggesting this threshold over-split actual diversity, though some MOTUs (23%) matched more than one species. It is not clear what (or if) an appropriate threshold would be. An intensive study of regional insect diversity is required to establish whether a MOTU approach can correctly approximate insect diversity in this region. In most of the cases (18 out of 23%) it was possible to identify the consumed species, because the rest of the species were not present in Western Europe. We found two MOTUs matching *Agrotis simplonia*, but if the first one was exclusive of this species, the second one matched other moth species as well. The only MOTU we assigned to *Hadena compta* matched several other species as well, some of them also present in Western Europe. As *Hadena compta* was the only species that matched at 100%, species-level identification was accepted. Razgour et al. [72] reported similar phenomena, showing the high similarity among sequences belonging to different species and a potential false positive using a short amplify sequence region. Consequently, it is advisable to use very conservative identification-criteria when assigning MOTUs to species.

Eight MOTUs we obtained from faeces did not match any sequence in the available reference databases. Although we can say they belong to 8 different species, we could not identify them. Nevertheless, these had likely very weak presence in the diet (4.3% of sequences), and are probably rare species limited to some high-mountain areas, which have not yet been sampled for barcoding purposes.

Correct identification of species may be conditioned by the presence of nuclear pseudogenes of the target sequence of DNA barcodes [113], [114]. Although several authors have demonstrated the presence of numts in insect taxa [114], we did not find any clear sign of nuclear pseudogenes. We took the measures proposed by Zhang & Hewitt [115] to detect numts, but no evidences such as suspicious mutations (indels, frame-shift mutations, or in-frame stop codons) or phylogenetic incongruences were detected. Even so, it is sometimes difficult to differentiate them from their mitochondrial paralogues [113], so their presence cannot be completely ruled out.

The individualised accumulation curves analysing 20 sequences per individual show that our results covered a great part of the specific diversity in diet for the sampling season. According to those curves, 100% of the estimated number of species was detected in 22 individuals. In the remaining 7 individuals, we likely did not uncover all prey content in the faeces. Lack of reliability of nonparametric species-richness estimators did not make it advisable to calculate the total species, especially when sample size was as small as in our case [116]. Therefore, the number of species that remained hidden in each dropping sample was not estimated. Nevertheless, the number of clones chosen in this study is balanced in terms of cost-efficiency, as all the estimated species were presumably detected in 72% of samples, and many more clones would have been required to produce a small increase in the number of species detected. High throughput pyrosequencing overcomes this constraint produced by clone numbers [72], but its cost/effectiveness depends on the objectives of the study, the economical constraints and the technical facilities available. Both methods are useful to detect the main bulk of species, but pyrosequencing may allow detecting also species with very low occurrence [72].

### Conclusions

The molecular analysis of diet, and particularly DNA mini-barcodes, has proven a highly useful tool to obtain an accurate description of the trophic resources used by animals, and also to unveil many aspects of their foraging behaviour based on the ecology of the prey species. This may be extremely helpful when dealing with elusive and hard-to-track species such as bats, or when implementation of non-invasive techniques is necessary or advisable due to conservation or ethical constraints. In our study, results show that the Mountain Long-eared Bat *P. macrobullaris* is a moth specialist that forages in high mountain meadows and rocky areas during summer. However, our data are restricted to summer and we should not dismiss the possibility that *P. macrobullaris* forages at lower elevations and in different habitats during other seasons, especially spring, when alpine habitats are moth-impoverished. Further research will be needed to unravel such questions. Moreover, detailed inspection of the diet content revealed that *P. macrobullaris* feeds mainly on tympanate moths, without any data supporting it hunts by gleaning prey. Thus, we conclude that the Mountain Long-eared Bat forages by aerial hawking, emitting faint echolocation pulses to avoid early detection by moths.

## Supporting Information

Figure S1 **The Mountain Long-eared Bat,** ***Plecotus macrobullaris*** **(photo A. Alberdi).**
(TIF)
