# Supplementary figures and images for: The Foraging Ecology of the Mountain Long-Eared Bat Plecotus macrobullaris Revealed with DNA Mini-Barcodes
Source: PLoS One. 2012 Apr 24;7(4):e35692. doi: 10.1371/journal.pone.0035692 (PMC3335802; doi:10.1371/journal.pone.0035692)

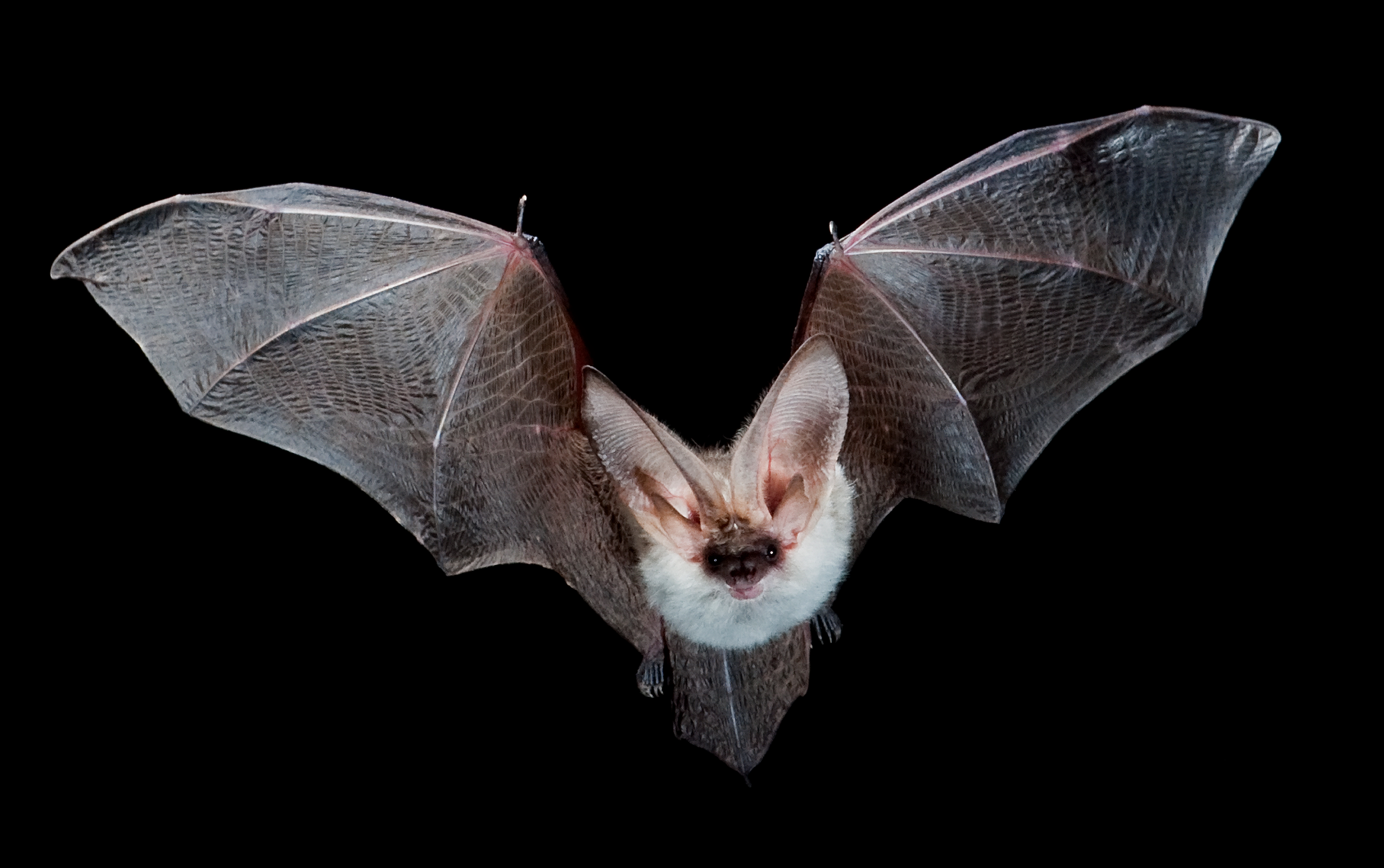

Supplement: Figure S1 — The Mountain Long-eared Bat, Plecotus macrobullaris (photo A. Alberdi). (TIF) [file pone.0035692.s001.tif]
